# Neuroinflammation and glial cell activation in mental disorders

**DOI:** 10.1016/j.bbih.2019.100034

**Published:** 2019-12-27

**Authors:** Priscila G.C. Almeida, João Victor Nani, Jean Pierre Oses, Elisa Brietzke, Mirian A.F. Hayashi

**Affiliations:** a Departamento de Farmacologia, Escola Paulista de Medicina (EPM), Universidade Federal de São Paulo (UNIFESP), São Paulo, SP, Brazil; b Programa Multicêntrico de Pós-Graduação em Bioquímica e Biologia Molecular, Instituto de Biociências, Universidade Federal do Mato Grosso do Sul, Campo Grande, MS, Brazil; c Department of Psychiatry, Queen’s University School of Medicine, Kingston, ON, Canada

**Keywords:** Inflammation, Mental disorders, Glia, Microglial activation, Schizophrenia, MD, mental disorders, SCZ, schizophrenia, BD, bipolar disorder, MDD, major depression disorder, PRRs, pattern recognition receptors, TLRs, toll-like receptors, CLRs, C-type lectin receptors, NF, necrosis factor, IRF, interferon regulatory factor, APCs, antigen presenting cells, IL, interleukin, TNF, tumor necrosis factor, CNS, central nervous system, CCL, C–C motif chemokine ligand, CXCL, X–C motif chemokine ligand, IFN, interferon, TGF, tumor growth factor, CSF, cerebrospinal fluid, IDO, indolamine 2,3-dioxygenase, KYNA, kynurenic acid, NMDA, N-methyl-D-aspartate, AMPA, alpha-amino-3-hydroxy-5-methyl-4-isoxazolepropionic acid, PPI, prepulse inhibition, NMR, nuclear magnetic resonance, MRI, magnetic resonance imaging, QUIN, quinolinic acid, α7-nAchR, alpha 7 nicotinic acetylcholine receptor, BBB, blood-brain barrier

## Abstract

Mental disorders (MDs) are highly prevalent and potentially debilitating complex disorders which causes remain elusive. Looking into deeper aspects of etiology or pathophysiology underlying these diseases would be highly beneficial, as the scarce knowledge in mechanistic and molecular pathways certainly represents an important limitation. Association between MDs and inflammation/neuroinflammation has been widely discussed and accepted by many, as high levels of pro-inflammatory cytokines were reported in patients with several MDs, such as schizophrenia (SCZ), bipolar disorder (BD) and major depression disorder (MDD), among others. Correlation of pro-inflammatory markers with symptoms intensity was also reported. However, the mechanisms underlying the inflammatory dysfunctions observed in MDs are not fully understood yet. In this context, microglial dysfunction has recently emerged as a possible pivotal player, as during the neuroinflammatory response, microglia can be over-activated, and excessive production of pro-inflammatory cytokines, which can modify the kynurenine and glutamate signaling, is reported. Moreover, microglial activation also results in increased astrocyte activity and consequent glutamate release, which are both toxic to the Central Nervous System (CNS). Also, as a result of increased microglial activation in MDs, products of the kynurenine pathway were shown to be changed, influencing then the dopaminergic, serotonergic, and glutamatergic signaling pathways. Therefore, in the present review, we aim to discuss how neuroinflammation impacts on glutamate and kynurenine signaling pathways, and how they can consequently influence the monoaminergic signaling. The consequent association with MDs main symptoms is also discussed. As such, this work aims to contribute to the field by providing insights into these alternative pathways and by shedding light on potential targets that could improve the strategies for pharmacological intervention and/or treatment protocols to combat the main pharmacologically unmatched symptoms of MDs, as the SCZ.

## Highlights

• Alterations in the cytokine profile in mental disorders patients.
• Unbalanced kynurenine pathway leads to excitotoxicity and NMDA hypoactivation.
• Increased glutamate production leads to excitotoxicity.
• Microglial and astrocytic activation influences in neurotransmission pathways.

## 1 Introduction

Mental disorders (MDs) are characterized by clinically significant disturbances in cognition and behavior/emotion regulation, which all together reflect a brain dysfunction. MDs are one of the leading global causes of disability, and with recognized high prevalence (Kyu et al., 2018). Despite the clear genetic predisposition, the etiology of MDs is multifactorial, meaning that there is a combined contribution of genetic and environmental factors (Caspi and Moffitt, 2006). The oldest and most widely accepted hypothesis for the pathophysiology of MDs, such as schizophrenia (SCZ), bipolar disorder (BD), and major depressive disorder (MDD), is based on the dysregulation of the monoaminergic neurotransmission. For instance, the dopaminergic hyperfunction in mesolimbic pathways and hypofunction in mesocortical pathways were shown to explain several of the main symptoms as the general restricted emotional experience, hallucinations, deliriums, and cognitive deficits (Carlsson and Lindqvist, 1963). However, more recent data also indicate an important contribution of the hypofunction of glutamatergic (Kantrowitz and Javitt, 2012) and N-methyl-D-aspartate (NMDA) receptors (Snyder and Gao, 2013).

SCZ is currently among the most impactful and complex MDs, being also recognized as a neurodevelopmental disorder (Jablensky et al., 2017; Murray et al., 2017). Multiple genetic variants are expected to contribute to SCZ, although each one with only small effects (Luo et al., 2019). Together they may represent the potential targets for the environmental risk factors as, for instance, the maternal infections (Weber-Stadlbauer, 2017) or immune activation (Bergdolt and Dunaevsky, 2019), which may be equally capable of affecting the neurodevelopment. Consistently, the neurodevelopmental hypothesis for SCZ conciliates the interaction between the genetic and environmental factors as the possible origin of aberrant process (es) which could affect early brain formation during the embryonic development, potentially explaining the increased risk of developing SCZ (Zwicker et al., 2018; Bergdolt and Dunaevsky, 2019).

SCZ has also been linked to inflammation as the hypothesis of maternal immune activation, since a risk factor for the progeny to develop this disorder in adulthood was previously accepted by many (Mongan et al., 2019). In fact, blood analysis revealed consistent increase of IL-6, IL-12, IL-1β, tumor necrosis factor alpha (TNF-α), and interferon gamma (IFN-γ) in patients with SCZ, as well as decreased IL-10 in relapsed SCZ inpatients; similarly as observed in the cerebrospinal fluid (CSF) of first onset and acute relapsed patients with SCZ, normalized for the antipsychotic treatment (Miller et al., 2011). In addition, increased levels of midbrain immune-related transcripts observed in SCZ and in murine offspring after maternal immune activation may suggest that immune-related changes in SCZ extend to dopaminergic areas of the midbrain, and that maternal immune activation could be a possible contributing factor underlying these persistent neuroimmune changes, which also includes the activation of microglial and astrocytic cells (Purves-Tyson et al., 2019).

In BD patients, mood fluctuations between episodes of elevation (mania) and depression (recurrent or single), interspersed with periods of euthymia, are classically reported (Grande et al., 2016), and their association with aberrant calcium and glutamate signaling was suggested to explain these phenotypes (Nurnberger et al., 2014). The involvement of the neuroimmune system in the pathological process of BD is also recognized (Niu et al., 2019).

Patients with MDD are characterized by depressed mood and/or loss of interest or pleasure in almost all regular activities, as well as by the presence of other symptoms, including changes in sleep patterns, restlessness, slow motor function, unwanted substantial weight changes, fatigue, loss of energy, feelings of worthlessness or guilt, loss of cognition and memory, among others. The efficacy of medications targeting monoaminergic pathways reinforces the involvement of these systems in the pathophysiology of MDD, although the lack of effectiveness in about one third of the cases may suggest that depression pathophysiology may go beyond monoamines (Chávez-Castillo et al., 2019; Pitsillou et al., 2019). New strategies, such as the use of rapid-action antidepressant ketamine, that targets, among others, the glutamatergic system, indicate the need to modulate other pathways to obtain real responses, especially in the so-called treatment resistant depression (Taylor et al., 2019).

Despite the incomplete knowledge about the pathophysiology of these mental conditions, different studies pointed out to the possible important roles of inflammation in MDs. In fact, a correlation between the peripheral immune modulators and psychiatric symptoms has long been demonstrated in either clinical or animal models (Mosley, 2015; Jeon et al., 2018). For instance, immunological alterations leading to inflammation mediators increases have been observed in several most common MDs, as a result of differential microglial activation (Barichello et al., 2019). Therefore, further understanding of inflammation in MDs, as well as how it correlates to the known alterations in each disease may open new opportunities for a more suitable and satisfactory treatment/intervention, occasionally with the power to contribute to the prevention as well.

## 2 Inflammation and neuroinflammation overview

### 2.1 Inflammation

Inflammation was conceived as the immune system defensive response of an organism against pathogens and/or tissue insults, mainly based on the presence of different pattern recognition receptors (PRRs). PRRs belong to membrane-bound receptor families, which include the Toll-like receptors (TLRs) and C-type lectin receptors (CLRs), in addition to various cytosolic sensors, that once in contact with specific pathogenic proteins, as pathogen- or danger-associated molecular patterns, activate different signaling pathways aiming to isolate, dilute or destroy the noxious agent (Kumar, 2019). For instance, TLRs recognize most bacterial and viral pathogenic threats, and initiate the signaling cascades that culminate in the cell recruitment and production of inflammation mediators, as cytokines and chemokines (Kawasaki and Kawai, 2014). This response initiates with neutrophilic extravasation to the parenchymal tissue, followed by the recruitment of monocytes and lymphocytes (B- and T-cells) (Oo et al., 2010). Dendritic cells, also known as antigen-presenting cells (APCs), present these antigens to naïve T- and B-cells, subsequently, activating them. This process induces the formation of various T-cells subpopulations with different functions in the inflammatory response (Taniuchi, 2018). Once activated, monocytes, lymphocytes, mast cells and macrophages induce further production of inflammatory cytokines as IL-1β, IL-6, and TNF-α, which are able to modulate the immune response triggered by the infection and/or inflammation, besides regulating the inflammation by itself (Turner et al., 2014). Activated macrophages are specifically divided in two subpopulations, namely M1 and M2, in which M1 macrophages have pro-inflammatory roles (pro-inflammatory cytokine production and pathogen elimination), while M2 macrophages have anti-inflammatory properties (production of IL-10) (Murray and Wynn, 2011).

### 2.2 Neuroinflammation

Neuroinflammation refers to the inflammation in the CNS, and several evidences have shown a two-way communication between the neuronal and immunological cells, enabling the cooperation at the hybrid junction level, which modulates the synaptic plasticity and neuroimmunity. In fact, inflammatory signaling exists in different ways in accessing the brain. For instance, the cytokines can cross the blood brain barrier (BBB) through circumventricular organs and specific saturable BBB transporters (Limanaqi et al., 2019). After an insult, the CNS activates the glial cells, especially the microglia, and a complex neuroinflammatory signaling pathway is initiated, leading to the production and release of diverse chemokines and cytokines (Carson et al., 2006).

Microglia are the macrophage-like resident cells in the CNS, where they are responsible for the recognition of pathogen and production of cytokines and chemokines, such as the pro-inflammatory mediators IL-1β, IL-6, TNF-α, CCL2 (C–C motif chemokine ligand 2), CCL5, CXCL1 (C-X-C motif chemokine ligand 1), nitric oxide (NO) and prostaglandins (DiSabato et al., 2016). Like the peripheral macrophages, in response to different stimuli, microglia can also be activated into two states, namely M1 (pro-inflammatory) and M2 (anti-inflammatory) (Boche et al., 2013).

The M1 activation is a response to the higher levels of IFN-γ and TNF-α, produced by natural killer cells and T-helper 1 lymphocytes, which results in increased release of pro-inflammatory cytokines as IL-β, IL-6, TNF-α, IL-23, and oxygen free radicals, as well as in increased phagocytic activity and antigen presentation. On the other hand, the M2 activation stimulates the release of anti-inflammatory cytokines as IL-10 and tumor growth factor beta (TGF-β), and this activation can inhibit the inflammatory phagocytosis, besides inducing tissue repair/wound healing (Mosser and Edwars, 2008). Microglial activation has a physiological role in cytokine production in early brain development and in adult CNS, as well as it also participates in immunological activities and synapses pruning (Salter and Beggs, 2014). On the other hand, aged microglia are more responsive to stimuli, and produce larger amounts of pro-inflammatory cytokines that can result in persistent neuroinflammation relevant to diverse neuropathological diseases (Wolf et al., 2017). Apart from aging, stress-associated microglial activation, primarily in the M1 state, could be directly correlated to depressive behaviors and anxiety (Zhu et al., 2019). In this case, the elevated levels of glucocorticoids and catecholamines promote the brain inflammation, with consequent release of IL-1β and other pro-inflammatory cytokines from the microglia which, in turn, can stimulate the glucocorticoid release by activating the hypothalamic-pituitary-adrenal axis (Sun et al., 2017).

Peripheral increases of inflammatory mediators in MD patients (for instance, in SCZ, BD and MDD) have suggested an association between the chronic inflammation and mental conditions (Niu et al., 2019). In addition, increased levels of pro-inflammatory cytokines and chemokines in the CNS, as a result of microglial activation, can contribute to specific characteristics and symptoms of different MDs (Lurie, 2018), as they may also interfere with relevant signaling pathways in each of these diseases (Haroon et al., 2018; Lanz et al., 2019).

The kynurenine and glutamate signaling pathways, as well as the alterations resulting from the microglial activation, and how they are associated to MDs will be further discussed as follow.

## 3 Kynurenine pathway

The kynurenine pathway metabolites interact with the glutamatergic and cholinergic receptors, namely NMDA and alpha 7 nicotinic acethylcholine (α7-nAch) receptors, respectively, and alterations in these glutamatergic/cholinergic pathways were suggested to be relevant to several MDs (Erhardt et al., 2017). In fact, the kynurenine pathway is the main responsible for the production of several neuroactive molecules, including serotonin, quinolinic acid (QUIN), kynurenic acid (KYNA), among others, which are generated by the degradation of the non-essential amino acid tryptophan and other substrates (Ruddick et al., 2006; Carvalho et al., 2017). In the CNS, most cell types, including neurons, microglia and even infiltrating macrophages, produce all enzymes associated with the kynurenine pathway, while the astrocytes and oligodendrocytes do not produce some of these enzymes and, consequently, are unable of synthesizing QUIN (Schwarcz and Stone, 2017).

### 3.1 Kynurenine pathway and MDs

QUIN is an endogenous NMDA agonist, whereas KYNA is a NMDA antagonist. Higher concentrations of both were previously reported in the brain during fetal development, which quickly declined after birth, indicating a possible relationship between the kynurenine metabolism and the development of CNS (Notarangelo and Pocivavsek, 2017). Moreover, NMDA receptors were demonstrated to be involved in neuronal migration, synapse formation, neurite outgrowth, and other events related to neuronal plasticity, and therefore, abnormal NMDA activation or inhibition could then compromise its functioning with consequent relevant brain and behavior alterations (Waters and Machaalani, 2004).

**Fig. 1:**
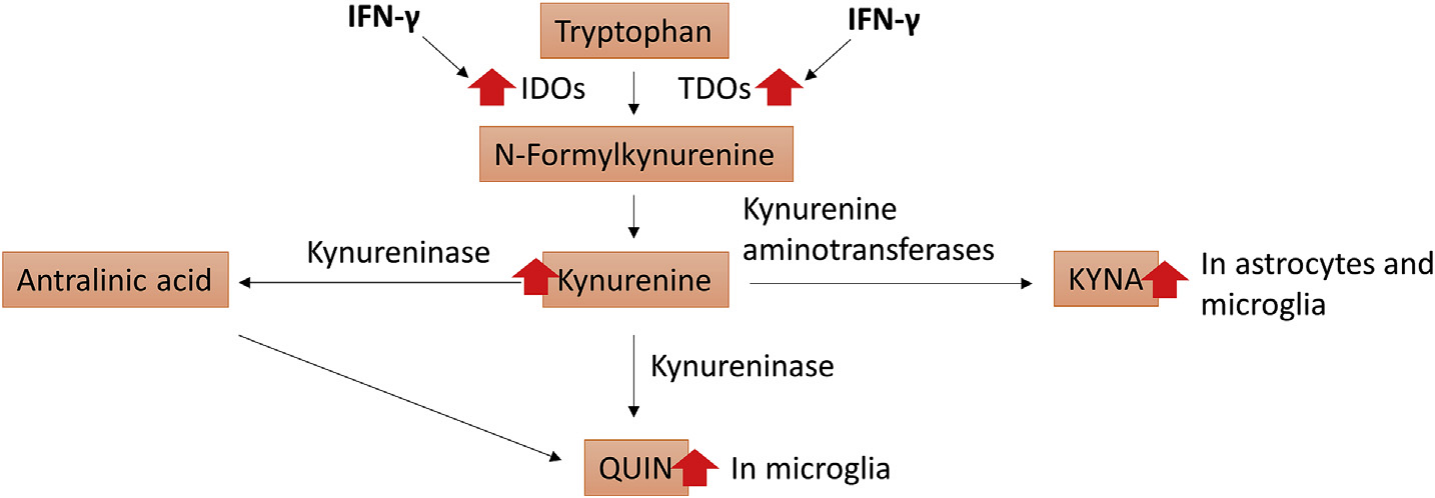
Schematic representation showing the kynurenine pathway and its neuroactive metabolites QUIN and KYNA. IFN-γ stimulates the activity of IDOs and TDOs in converting tryptophan into kynurenine, which crosses the BBB and is converted into QUIN in the microglia, and KYNA in both microglia and astrocytes.

Increased pro-inflammatory cytokines, as a result of immune activation, can modulate the kynurenine pathway in the CNS, as IFN-γ stimulates the production of indolamine 2,3-dioxygenases (IDOs) and tryptophan 2,3-dioxygenase (TDOs) (Kim and Jeon, 2018) (Fig. 1). Kynurenine can cross the BBB, and once in the CNS it is absorbed by the glial cells, and subsequently metabolized into KYNA and QUIN (Kennedy et al., 2017), with different consequences, depending on the levels of each metabolite generated. KYNA is often described as neuroprotective in the brain, with sedative and anticonvulsant effects at low concentrations, while QUIN is described to be neurotoxic, inducing apoptosis in Th1 target cells and selectively inhibiting the proliferation of natural killer cells, namely CD4^+^ and CD8^+^ T-lymphocytes, once these cells are in their active state (Chen and Guillemin, 2009).

During the inflammatory response, IDO-1 and IDO-2 are upregulated by pro-inflammatory cytokines (Campbell et al., 2014), resulting in excessive production of KYNA and QUIN, which can both determine the neurotoxic effects mediated by the NMDA receptor (Schwarcz et al., 2012), and also, by indirectly altering other signaling pathways, including the dopaminergic pathway (Erhardt et al., 2017).

SCZ and BD were both previously associated with inflammation and high levels of pro-inflammatory cytokines, and increased levels of KYNA in the CSF and *post-mortem* brain of patients with SCZ and BD have been consistently described (Schwieler et al., 2015; Kindler et al., 2019). However, QUIN levels in SCZ and BD are still a matter of contradiction in the literature. While some studies indicate that the levels of QUIN and its precursor 3-hydroxykynurenine are unaltered in CSF and *post-mortem* brain of BD and SCZ patients (Sathyasaikumar et al., 2011; Kegel et al., 2014), some others have suggested elevated peripheral levels of 3-hydroxykynurenine, which were apparently normalized after the treatment with antipsychotic drugs (Oxenkrug et al., 2016). Alterations in the KYN/tryptophan relation, as well as increased level of QUIN and KYNA after a second immune challenge in early adulthood, were observed in animal models for maternal immune activation (Clark et al., 2019).

In addition, in contribution to the theory of NMDA receptor hypofunction in SCZ (Snyder and Gao, 2013), a study with *post-mortem* samples of hippocampus of SCZ patients has demonstrated decreased contents of the NMDA receptor agonist QUIN in microglia, with levels even lower during the psychotic episode (Gos et al., 2014).

## 4 Glutamate pathway

Glutamate is an endogenous amino acid that acts as an excitatory neurotransmitter in the CNS, with a suggested important role in learning and memory, and in neuronal plasticity, by modulating cell elimination and neuronal development (McEntee and Crook, 1993). The neurotransmitter glutamate interacts with different types of receptors as, for instance, the NMDA ligand-gated ion channels and AMPA (alpha-amino-3-hydroxy-5-methyl-4-isoxazolepropionic acid) ionotropic receptors (Ribeiro et al., 2017). Its roles in cell proliferation, neuronal migration, synaptic transmission, excitability, plasticity, amongst others, are well established (Moretto et al., 2018), allowing therefore the association of this signaling pathway with a distinct set of disorders, including neurodevelopmental and neurodegenerative psychiatric conditions (Ribeiro et al., 2017; Moretto et al., 2018). This important component to brain health needs a strict signaling regulation, as excessive glutamate can cause cell death in a process referred as “excitotoxicity”, which occurs after activation of glutamate receptors in brain cells (Zhou and Danbolt, 2014).

Besides the increased release of pro-inflammatory mediators, microglial activation also results in decreased expression of glutamate transporters and receptors, stimulating increased production and release of glutamate in microglia (Haroon et al., 2017). Moreover, TNF-α also induces glutamate release from microglia and astrocytes (Tasker et al., 2012). Inflammatory mediators, such as chemokines and cytokines, produced either by the activated microglia in the CNS or by the peripheral macrophages in response to an immune response, activate astrocytes and stimulate the production of reactive oxygen species and glutamate release (Haroon and Miller, 2017).

Although the mechanism by which glutamate is correlated to MDs is not completely clear yet, it is known that glutamate is released by astrocytes and neurons in the CNS, in a process involving microglia and oligodendrocytes (Gundersen et al., 2015).

### 4.1 Glutamatergic signaling and MDs

Various MDs such as MDD, SCZ and post-traumatic stress disorder, are considered to be glutamate-related, especially regarding the interaction with the NMDA receptors (Schwarcz and Stone, 2017; Haroon and Miller, 2017). Interestingly, studies have suggested that the dopaminergic alterations observed in SCZ are mediated by altered glutamatergic signaling, due to a NMDA hypofunction, even considering the assumed increased glutamate release during neuroinflammation (Schwartz et al., 2012). For instance, clinical studies with NMDA antagonists, as phencyclidine and ketamine, have demonstrated the presence of characteristics similar to most common symptoms of SCZ, such as the emotional blunting, thought disorders, hallucinations, cognitive deficits, anhedonia and social retreat (Krystal et al., 2005).

In animal models, NMDA hypofunction (induced either by pharmacological or genetic approaches) resulted in behavioral changes associated with SCZ symptoms, as for instance, the hyperlocomotion in the open field test and the deficits in prepulse inhibition (PPI) of acoustic startle response, which were both reversed by the administration of antipsychotic drugs, such as clozapine and haloperidol (Lee and Zhou, 2019).

The progressive loss of brain tissue reported in SCZ (Chew et al., 2013) could also be correlated to glutamatergic dysregulation during the developmental period, since it could also affect the NMDA roles in synaptic plasticity and neuronal activity (Harrison and Weinberger, 2005). Neonatal administration of NMDAR antagonist phencyclidine in rats induced functional deficits in hippocampal brain network, as denoted by the changes in oscillatory rhythms in the excitation/inhibition balance of the brain (Kjaerby et al., 2017).

Regardless of the possible correlation of glutamate with SCZ, most of data found in the literature refer to the glutamatergic contribution in MDD, in which the association with microglial activation and consequent neuroinflammation was early demonstrated (Leonard, 2018). In fact, depressive patients presented high glutamate levels in the plasma, CSF and brain (Sanacora et al., 2008). In addition, it is likely that glutamate signaling alterations in the brain are region-dependent, as decreased levels of glutamate in the prefrontal cortex and anterior cingulate cortex were noticed by nuclear magnetic resonance (NMR) spectroscopy studies (Murrough et al., 2017). In addition, the NMDA receptor antagonist ketamine showed antidepressant effects in patients, as suggested by the reduction of sad mood, anhedonia, pessimism and indecision (Williams and Schatzberg, 2016), and also in experimental animal models, as demonstrated by several behavioral tests (Refsgaard et al., 2017).

Clinical evidence also points out to a key role of glutamatergic transmission in the pathophysiology of BD (Blacker et al., 2017). Neurophysiological abnormalities, related to the glutamate-glutamine cycle, membrane turnover, and neuronal integrity, have also been described in BD patients (Kubo et al., 2017). Literature data regarding glutamate/NMDA alterations in BD patients in depressive phase are in accordance with those for depressive patients, mainly concerning the effects of the antidepressant ketamine (Zarate et al., 2012). Studies with BD patients demonstrated links between the genes responsible for coding ionotropic glutamate receptors subunits, the risk for BD, and the response to the treatment with lithium (Le-Niculescu et al., 2009). Lithium has been shown to decrease both glutamatergic and dopaminergic excitatory signaling (Malhi et al., 2013), while proton NMR spectroscopy studies in BD patients revealed elevated prefrontal glutamate across mood states (Smaragdi et al., 2019).

## 5 Dopamine pathway

Dopamine is a catecholaminergic neurotransmitter that plays an important role in motor function, motivation, cognition, emotion and neuroendocrine secretion. Dopaminergic innervation is the most prominent in the brain, and the four major signaling pathways identified are the nigrostriatal, mesolimbic, mesocortical and tuberoinfundibular (Beaulieu and Gainetdinov, 2011). Dopamine is early expressed in developing brain and it is relevant to the development of neuronal cytoarchitecture, with influence in processes such as the cell proliferation, migration and differentiation (Money and Stanwood, 2013). Therefore, changes in this pathway can lead to altered connectivity and dysfunctional synapses, as demonstrated in animal models with disrupted dopaminergic signaling (Zhang et al., 2010). From the five known dopamine receptors (D1, D2, D3, D4 and D5), three were implied to contribute to SCZ symptoms, with the negative symptoms resulting from reduced D1 activation and possible D3 alterations, and with the positive symptoms resulting from increased D2 expression (Simpson et al., 2014).

### 5.1 Dopamine system and MDs

The impact of dopaminergic transmission in SCZ is widely studied, since most antipsychotic drugs relief symptom by interacting directly or indirectly with dopamine receptors D2 (Amato et al., 2018). The dopamine receptors of mesolimbic and mesocortical pathways are the main targets of the antipsychotic drugs currently used for the treatment of several MDs, and increased dopaminergic signaling in the mesolimbic is described to be responsible for the positive symptoms observed in SCZ, while decreased dopaminergic signaling in the cortex is suggested to be responsible for the negative symptoms (McCutcheon et al., 2019).

The mechanisms related to the dopamine neurotransmission and symptoms in BD are similar to that in SCZ (Ashok et al., 2017), in which increased signaling during the mania phase and a decreased signaling during the depressive phase are reported (Tye et al., 2013; Sidor et al., 2015). Moreover, dopamine antagonists and/or partial agonists are often used in the treatment of acute mania, bipolar depression and also for treatment maintenance (Hooshmand et al., 2014). Moreover, dopaminergic transmission seems to be mildly modulated by glutamate and other neuroactive metabolites capable of interacting with NMDA and AMPA receptors (Felger, 2017).

Deficits in the reward process, motivation and motor function are commonly described in MDs, and a correlation between these symptoms and dopaminergic dysfunction and inflammation is discussed (Felger and Treadway, 2017). Even if the mechanism by which this signaling is altered is still not clear, there is evidence supporting that activation of innate immunity and release of inflammatory cytokines may both play important roles (Harrison et al., 2015), by affecting the dopamine synthesis, release and uptake, in order to reduce the dopamine neurotransmission in the basal ganglia (Felger, 2017). The effect in synthesis is due to increased release of QUIN, KYNA and glutamate, that contribute together to oxidative stress and generation of reactive oxygen species, ultimately leading to decreased production of L-3,4-dihydroxyphenylalanine (L-DOPA) (Vancassel et al., 2018). Besides, KYNA is a NMDA antagonist, and therefore, could negatively modulate the dopamine release (Giménez-Gómez et al., 2018). Increased cytokines could decrease the expression or function of the vesicular monoamines transporter 2 (VMAT2), and can increase the dopaminergic reuptake by the dopamine transporter (DAT), ultimately leading to altered dopaminergic signaling, as it also occurs with serotonin transporters (van Heesch et al., 2013).

## 6 Conclusion or final remarks

Although there are recommended treatments for MDs, many of them do not show efficacy in all patients or do not necessarily relieve all symptoms, besides showing several undesirable side effects. For this reason, it is important to keep investigating the underlying pathophysiology aiming to discover and characterize new targets for pharmacological intervention.

**Fig. 2:**
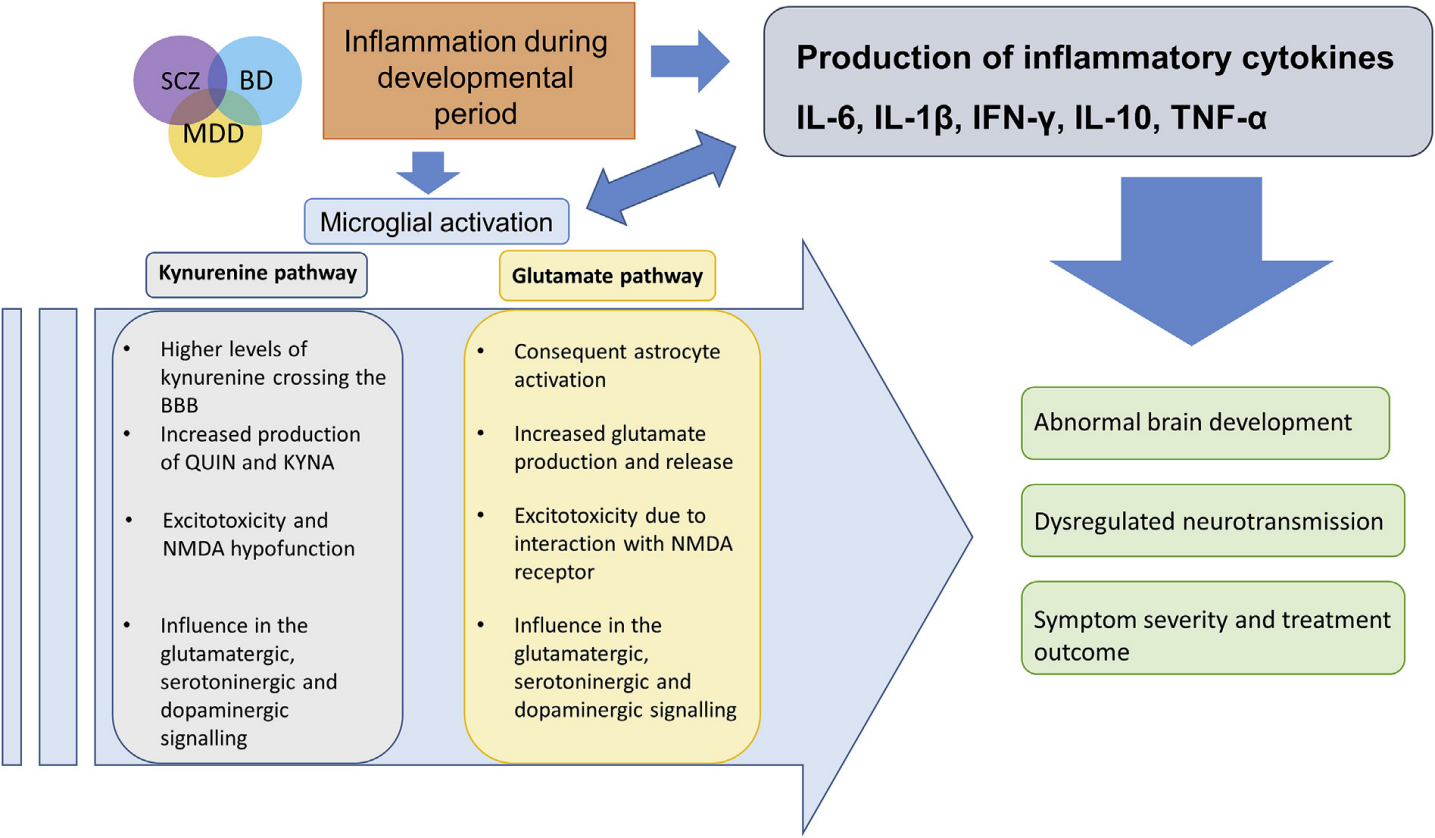
MDs such as SCZ, BD and MDD, that share symptoms and aspects of their pathophysiology, have been wildly associated to immune activation during developmental period. In this case there is an increased production of inflammatory cytokines, both in the periphery and in the CNS, the latter by activated microglia. Microglial activation also results in increased production of the kynurenine pathway neuroactive metabolites QUIN and KYNA, which results in excitotoxicity and NMDA hypofunction. Besides, activated microglia stimulates astrocytic activation, which in turn increases glutamate release with consequent excitotoxicity. Alterations in QUIN, KYNA and glutamate levels can influence the glutamatergic, serotoninergic and dopaminergic signaling, and together with the increased inflammatory cytokines, result in abnormal brain development and dysregulation in neurotransmissions, ultimately affecting symptom severity and treatment outcome.

Microglial activation was shown to modulate the release of glutamate and production of metabolites from the kynurenine pathway, which are capable of modulating the glutamatergic, serotoninergic and dopaminergic signaling, mainly through interaction with NMDA glutamatergic receptors (Fig. 2). These receptors were demonstrated to be altered in a distinct set of MDs, and to contribute to some of reported/observed symptoms. Since it is known that MDs are influenced by environmental factors, besides the importance of the genetic predisposition, the immune activation may emerge as the missing link between these two factors.

Therefore, new treatment possibilities may emerge by interfering not only in the classic monoaminergic signaling pathways, but also targeting upstream in the cascade of events that results in these well-known alterations in the monoaminergic signaling. In this context, early intervention in the production of the inflammatory cytokines, kynurenine pathway metabolites and glutamate, or in their interactions with receptors, may have the power to decrease the effects of other relevant monoaminergic pathways responsible for the MDs symptoms, including the dopaminergic, serotoninergic and glutamatergic signaling. We recognize that further experimental data are essential to solidify this suggestion, but this work aimed mainly to contribute to summarize how these altered pathways are correlated to MDs. In addition, we would like to stimulate further studies in the field by shedding light on the potential new possibilities for pharmacological intervention for the treatment of unmet symptoms in MDs.

## Financial support

This work was supported by the 10.13039/501100001807Fundação de Amparo à Pesquisa do Estado de São Paulo [Grant No: 2014/50891-1, 2017/02413-1 and 2018/20014-0]; and the 10.13039/501100003593Conselho Nacional de Desenvolvimento Científico e Tecnológico [Grant No: 454234/2014-7 and 455953/2014-7]. This study was also financed in part by the 10.13039/501100002322Coordenação de Aperfeiçoamento de Pessoal de Nível Superior - Brazil - Finance Code 001.

## Declaration of competing interest

The authors declare no conflicts of interest.
